# Selective Cytotoxicity of Complexes with N,N,N-Donor Dipodal Ligand in Tumor Cells

**DOI:** 10.3390/ijms22041802

**Published:** 2021-02-11

**Authors:** Malgorzata Tyszka-Czochara, Anna Adach, Tomasz Grabowski, Paweł Konieczny, Paweł Pasko, Joanna Ortyl, Tomasz Świergosz, Marcin Majka

**Affiliations:** 1 Faculty of Pharmacy, Jagiellonian University Medical College, 30-688 Krakow, Poland; paskopaw@poczta.fm; 2 Institute of Chemistry, Jan Kochanowski University, 25-406 Kielce, Poland; anna.adach@ujk.edu.pl; 3 Polpharma Biologics S.A., 80-172 Gdańsk, Poland; tomasz.grabowski@PolpharmaBiologics.com; 4 Faculty of Medicine, Jagiellonian University Medical College, 30-663 Krakow, Poland; pawel.konieczny@outlook.com (P.K.); marcin.majka@uj.edu.pl (M.M.); 5 Department of Biotechnology and Physical Chemistry, Faculty of Chemical Engineering and Technology, Cracow University of Technology, 31-155 Kraków, Poland; jortyl@pk.edu.pl; 6 Photo HiTech Ltd., Life Science Park, Bobrzyńskiego 14, 30-348 Cracow, Poland; 7 Department of Analytical Chemistry, Faculty of Chemical Engineering and Technology, Cracow University of Technology, 31-155 Kraków, Poland; tomasz.swiergosz@pk.edu.pl

**Keywords:** cytotoxicity, selectivity, cancer, VO^2+^, Co^2+^ complexes, N,N-bis(3,5-dimethylpyrazolyl-1-methyl)amine

## Abstract

The present article demonstrates selective cytotoxicity against cancer cells of the complexes [Co(L^D^)_2_]I_2_∙CH_3_OH (1), [CoL^D^(NCS)_2_] (2) and [VOL^D^(NCS)_2_]∙C_6_H_5_CH_3_ (3) containing the dipodal tridentate ligand LD = N,N-bis(3,5-dimethylpyrazol-1-ylmethyl)amine), formed in situ. All tested complexes expressed greater anticancer activities and were less toxic towards noncancerous cells than cisplatin. Cobalt complexes (1 and 2) combined high cytotoxicity with selectivity towards cancer cells and caused massive tumour cell death. The vanadium complex (3) induced apoptosis specifically in cancer cells and targeted proteins, controlling their invasive and metastatic properties. The presented experimental data and computational prediction of drug ability of coordination compounds may be helpful for designing novel and less toxic metal-based anticancer species with high specificities towards tumour cells.

## 1. Introduction

Recently, several reports have emphasized the use of transition metal complexes other than cisplatin as alternatives to existing standard approaches [1,2,3,4]. Growing evidence shows that some cobalt- or vanadium-containing compounds may be less toxic alternatives for platinum-based anticancer drugs [5]. These complexes might be used for the synthesis of new generations of safe and effective anticancer agents [5,6,7]. In particular, several metallodrugs [1] and coordination compounds [2,3,5,8,9,10,11,12] have been launched and examined. The identification of structural properties of metal-based complexes that may augment their antitumour activities is the sole basis of designing novel potent complexes. We have previously reported the one pot synthesis and physicochemical characterizations of complexes containing N,N,N-tris (3.5-dimethylpyrazol-1-ylmethyl)amine ligands (L^S^) [6,13,14,15,16], which is an analogue of original poly(pyrazol-1-yl) borates called scorpionates [17]. Complexes with N,N,N-tris(3.5-dimethylpyrazol-1-ylmethyl)amine ligands (L^S^) showed high antiproliferative activities against a panel of human cancer cells [6,15,16]. The nature of ligands and their architectures significantly determines the properties of resulting complex species [6,13,14,15,16,18,19,20,21]. We have also previously found that cationic–anionic (Co/Zn and Co/Cd) complexes containing N-scorpionate ligands (such as N,N,N-tris (3.5-dimethylpyrazol-1-ylmethyl)amine (L^S^)), express substantial antiproliferative potency [6,15]. The cytotoxic evaluation of these complexes also revealed their high selectivity for cancer cells. Moreover, all complexes were much more active against tumour cells and less harmful towards normal cells than the reference drug, cisplatin. As an extension of the above results, we decided to study anticancer activities of complexes containing the multipodal ligand N,N-bis(3.5-dimethylpyrazol-1-ylmethyl)amine (L^D^), an analogue of N,N,N-tris(3.5-dimethylpyrazol-1-ylmethyl)amine (L^S^). In particular, we aimed to find out whether the specific architectures of these complexes may influence the selectivity of investigated compounds. It is worth noting that biological activities of complexes containing N,N-bis(3,5-dimethylpyrazol-1-ylmethyl)amine (L^D^) have been poorly tested so far.

**Scheme 1:**
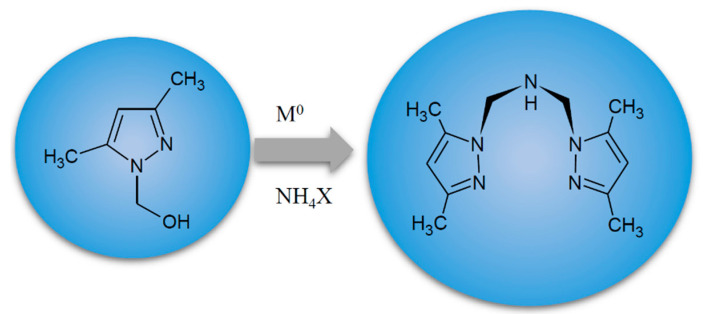
The route of formation of N,N-bis(3,5-dimethylpyrazol-1-ylmethyl)amine (L^D^) [16].

In this paper, we employed three complexes containing N,N,N-donor dipodal ligands (L^D^), forming anionic and neutral complexes with different geometries. For the purpose of biological study in this work, we prepared three coordination compounds—[Co(L^D^)_2_]I_2_∙CH_3_OH (1), [CoL^D^(NCS)_2_] (2) and [VOL^D^(NCS)_2_]∙C_6_H_5_CH_3_ (3)—containing the dipodal tridentate ligand L^D^ formed in situ from 1-hydroxymethyl-3,5-dimethylpyrazole (L^0^) as a substrate and precursor (Scheme 1) [6,15,16]. In particular, we aimed to evaluate the biological effects of complexes containing the N,N-bis(3,5-dimethylpyrazol-1-ylmethyl)amine (L^D^) ligand in cultures of tumour and noncancerous cells and correlate anticancer potency with crystalline and elemental compositions of isolated complexes formed in situ. It should be also noted that, so far, no complexes containing N,N-bis(3,5-dimethylpyrazol-1-ylmethyl)amine (L^D^) were investigated regarding specificity of action towards tumour cells. Interactions with tumour cell membrane accounts for biological properties of compounds and activities such as the ones investigated here have not yet been studied. The data obtained in an in vitro model and drugability assessment may be helpful for designing novel, less toxic anticancer coordination complexes with great specificity towards tumours. Such metal complexes may potentially be used as antitumour drugs or as adjuvants increasing efficiency of existing therapies.

## 2. Results

In this paper, we aimed to find out whether complexes 1, 2, and 3 can express selective cytotoxicity on cells in vitro. To achieve this goal, tumour cells (Hep G2) and noncancerous (CHO-K1) cells were employed and the ability of compounds to interact with the cell membrane and cancer cell regulatory proteins was investigated. The results of these studies are presented below.

### 2.1. Cobalt and Vanadium Complexes Exert Toxic Effect on Tumour Cells but Not in Noncancerous Cells

**Figure 1:**
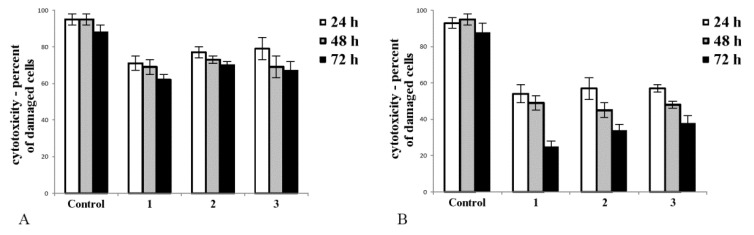
The cytotoxicity of complexes 1, 2, and 3 measured in noncancerous CHO-K1 cells (A) and tumour HepG2 cells (B) upon 24, 48, and 72 h of exposure (50 µM/L of each compound was added to the appropriate well). Trypan blue exclusion test was employed to distinguish between viable cells and cells with disrupted cell membranes. Note that all tested complexes were less cytotoxic toward noncancerous (A) as opposed to cancer cells (B). All tested complexes caused a statistically significant decrease in viable cells in a population of cells compared to control after 24 h exposure (normal cells: (A) *p* < 0.05 for 1 vs. control, *p* < 0.05 for 2 vs. control and *p* < 0.05 for 3 vs. control; tumour cells: (B) *p* < 0.01 for 1 vs. control, *p* < 0.01 for 2 vs. control and *p* < 0.01 for 3 vs. control). In cancer cell culture, 72 h exposition for all tested compounds caused a statistically significant toxic effect ((B), *p* < 0.01 for 1 vs. control, *p* < 0.01 *for 2* vs. control, and *p* < 0.01 *for 3* vs. control). Complex 1 caused the greatest decrease in cancer cell culture after 72 h exposure. In this study, 2 × 10^4^ cells/well was used to established cell culture. The number of viable and dead cells was measured using the automatic cell counter Countess (Gibco Laboratories, Grand Islands, NY, USA). The bars represent the arithmetic mean values and standard deviation (M; ±SD), *n* = 3.

We aimed to find out whether tested complexes affect the integrity of the cell membrane. Complexes 1, 2, and 3 had diverse effects in noncancerous (CHO-K1, Figure 1A) and cancer (Hep G2, Figure 1B) cell cultures. Upon treatment, the number of disrupted cells was greater in cancer cell culture (Figure 1B) than in culture of noncancerous cells (Figure 1A). In cultures of tumour cells, the toxicity of tested complexes significantly increased with time of exposure. The most toxic complex towards tumour cells was 1, and it caused the greatest decrease in Hep G2 cell viability (Figure 1B, after 72 h exposure).

### 2.2. Cobalt and Vanadium Complexes Hamper Growth of Cancer Cells

**Figure 2:**
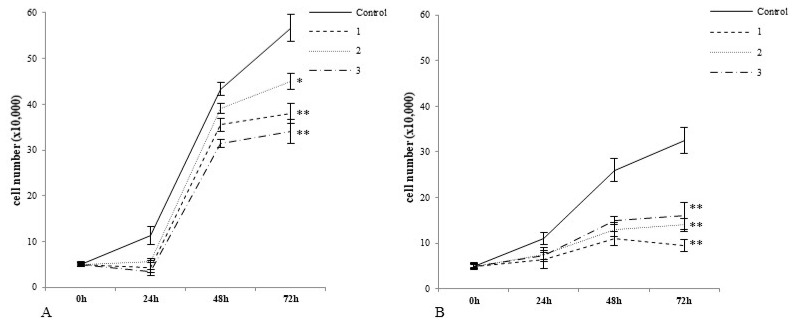
Complexes 1, 2 and 3 differentially regulate the growth of noncancerous CHO-K1 cells and tumour HepG2 cells. Note that complex 2 had the weakest inhibitory effect on normal cells after 72 h exposure (A). Complex 1 exerted the greatest inhibitory effect on tumour cells grown after 72 h treatment (B). In this study, 3 × 10^3^ cells/well was initially used to established cell culture. The number of cells at each time point was measured with the automatic cell counter Countess (Gibco Laboratories, Grand Islands, NY, USA). The plots represent arithmetic mean values and standard deviation (M; ±SD), *n* = 3; (* *p* < 0.05 vs. control, ** *p* < 0.01 vs. control).

In particular, we examined the potencies of complexes 1, 2, and 3 to decrease the growth of noncancerous cells and tumour cells upon treatment. As shown in Figure 2B, 72 h incubation with all tested complexes caused a decrease in cancer cell growth compared to control cells (Figure 2B). The greatest inhibitory effect was caused by 1, followed by 2 and 3. After 72 h incubation with normal cells, each of the tested compounds caused a statistically significant decrease in growth (Figure 2A). Here, complex 2 expressed the lowest inhibitory potency which changes in the following order, 1 > 3 >2.

### 2.3. Cobalt and Vanadium Complexes Exert Antiproliferative Effects on Tumour Cells

For the assessment of antiproliferative potency of complexes, an MTT assay was employed. The cell cultures were exposed for 72 h to synthesized cobalt and vanadium complexes or inorganic salts (CoCl_2,_ and VOSO_4_). An anticancer drug, cisplatin, was also used in the experiment. The IC_50_ values for each compound were calculated [6] and are presented in Table 1. The obtained results show that the IC_50_ values for all tested complexes were lower in cultures of cancer cells than in noncancerous cells. In tumour Hep G2 cells, complex 1 had the greatest antiproliferative effect when compared to 2 (Table 1; 22.0 vs. 38.2 μM) and 3 (22.0 vs. 45.6 μM). The inhibitory effect of 1 was comparable to the action of cisplatin (22.0 vs. 21.3 μM, respectively). In general, complexes 1, 2 and 3 were more active in culture of tumour cells than in noncancerous cells. In order to compare the specificity of compounds towards tumour cells, the Antiproliferative Index (AI) was calculated (see our previous study [6]) and presented in Table 1. Interestingly enough, even though 1 caused the greatest antiproliferative effect in Hep G2 cells (with IC_50_ value 22.0 μM), 2 was the most specific compound towards tumour cells, with minor cytotoxicity towards noncancerous cells (AI value for 2 was 7.0 vs. AI = 5.5 for 1 and AI = 2.7 for 3). It should be underlined that the AI value for 2 was 7-fold higher than the AI value for cisplatin (AI = 7.0 vs. AI = 0.9, respectively). As shown in Table 1, all synthesized complexes were more active and more specific towards cancer cells than appropriate comparative salts.

### 2.4. Cobalt and Vanadium Complexes Induce Apoptosis in Cancer Cells

**Figure 3:**
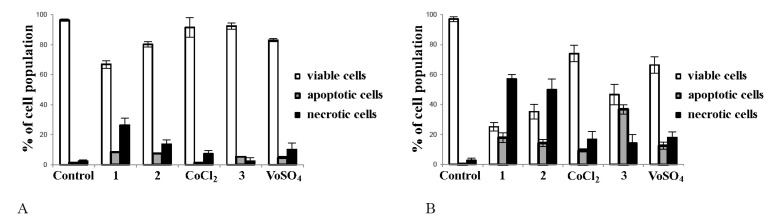
The effects of complexes 1, 2 and 3 on apoptosis/necrosis in CHO-K1 (**A**) and HepG2 cells (**B**) determined by flow cytometry. The cells (2 × 10^4^/well) were exposed to 50 μM/L of each compound for 24 h. In parallel, the cells were incubated in the same conditions with addition of comparator salts: CoCl2 (comparator for cobalt complexes 1 and 2) and VOSO_4_ (comparator for vanadium complex 3). Note that complex 3 was the most active promotor of apoptosis ((**B**), *p* < 0.001 vs. control) while complexes 1 and 2 caused mainly necrotic cell death ((**B**), for 1 and 2 *p* < 0.01 vs. control). Apoptosis was determined by flow cytometry with Annexin V/EthD-III double staining and DAPI as a control of viable cells. Three experiments were performed with similar results and the bars represent arithmetic mean values and standard deviation (M; ±SD), *n* = 3.

In the next step, we aimed to find out which mechanism of cell death was involved in the action of the synthesized complexes. To recognize, whether proapoptotic and/or pronecrotic activity is behind the anticancer action of tested compounds in tumour cells, flow cytometry analysis was employed. As presented in Figure 3, complexes 1, 2 and 3 elucidated cell death in tumour Hep G2 cells (Figure 3B), while in noncancerous CHO-K1 cells, the inhibitory effect of complexes was minor (Figure 3A). The data demonstrated that the exposition of cancer cells to vanadium complex caused the greatest apoptosis. The effect exerted for vanadium salt (VOSO_4_) was much weaker than complex 3 (Figure 3B, *p* < 0.05 for 3 vs. VOSO_4_). Cobalt complexes showed ability to initiate tumour cell death mainly due to necrosis. Both complexes 1 and 2 induced greater necrosis in tumour cells than their salts (CoCl_2_) (*p* < 0.05 for 1 vs. CoCl2 and *p* < 0.05 for 2 vs. CoCl_2_).

### 2.5. Cobalt and Vanadium Complexes Impair Expression of Genes Regulating Aggressive Phenotype of Cancer Cells

**Figure 4:**
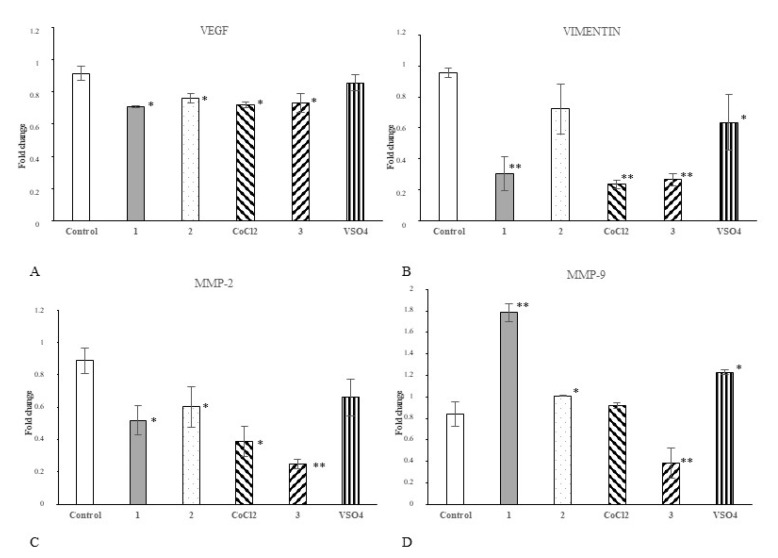
The effect of complexes 1, 2 and 3 and their salts on the expression of genes controlling angiogenesis and invasive/metastatic phenotype of tumour cells. The expression of mRNA for vascular endothelial growth factor a (VEGFa) (A), vimentin (Vim) (B), matrix metalloproteinase-2 (MMP-2) (C) and matrix metalloproteinase-9 (MMP-9) (D) in Hep G2 cells was analysed with Real-Time PCR (qPCR). The cells (2 × 10^4^/well) were exposed to 50 μM/L of each compound (or appropriate comparator salt) for 24 h. The data were normalized against reference gene Glyceraldehyde 3-phosphate dehydrogenase (GAPDH) transcript; the 2^−ΔΔCt^ method was used for determination of mRNA level; * *p* < 0.05 and ** *p* < 0.01 vs. untreated control). The real-time PCR data were presented as arithmetic mean values and standard deviation (M; ±SD), *n* = 3.

In the next step, we investigated if our cobalt and vanadium complexes may affect the expression of selected regulatory proteins in cancer cells. The changes of expression of appropriate genes were determined upon 24 h of Hep G2 cell treatment with each compound or appropriate salt comparator (50 μM/L). We focused on proteins determining angiogenic properties (vascular endothelial growth factor, VEGF-A), migratory phenotype (vimentin, Vim) and invasive/metastatic potential (Matrix metalloproteinases, MMP-2 and MMP-9) of cancer cells. Real-Time PCR (qPCR) analysis revealed that all tested complexes, 1, 2 and 3, significantly hampered VEGFa expression in Hep G2 cells (Figure 4A). Complexes 1 and 3 decreased Vim expression comparing to control (Figure 4B). Vanadium complex had the greatest inhibitory effect on the expression of matrix metalloproteinases MMP-2 (Figure 4C) and MMP-9 (Figure 4D). Both cobalt complexes decreased MMP-2 gene expression (Figure 4C). Similarly, comparator salt CoCl_2_ decreased mRNA for MMP-2, but did not changed transcript level for MMP-9 (Figure 4C,D).

### 2.6. Cobalt Complexes Increase Glutathione Peroxidase (Gpx) Activity in Noncancerous Cells and Vanadium Complex Decreases the Enzyme Activity in Cancer Cells

**Figure 5:**
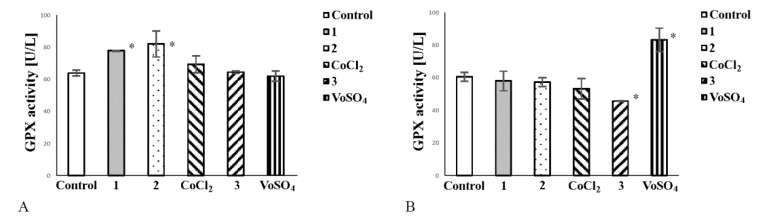
The effect of complexes 1, 2 and 3 on glutathione peroxidase (GPx) activity in culture of noncancerous cells CHO-K1 (**A**) and tumour HepG2 cells (**B**). The cells (2 × 10^4^/well) were exposed to 50 μM/L of each compound or comparator salt for 24 h. Note that complex 3 decreased GPx activity in cancer cells (**B**) and complexes 1 and 2 elevated the enzyme activity in noncancerous cells (**A**). The enzyme activity was determined spectrophotometrically. The bars represent arithmetic mean values and standard deviation (M; ±SD) (*n* = 3; * *p* < 0.05 vs. untreated control).

Most tumour cells efficiently manage oxidative stress. Glutathione Peroxidase (GPx) (EC 1.11.1.9) provides adequate protection of cells against damage caused by oxidative stress and may act as a prosurvival factor. Therefore, we assessed if tested complexes are capable of reducing activity of GPx. In a culture of noncancerous cells, both cobalt complexes increased GPx activity (Figure 5A). CoCl_2_ did not exert any effect on GPx activity in CHO-K1 cells (Figure 5A). Complex 3 decreased GPx activity in cancer cell culture (Figure 5B). VOSO_4_ elevated the enzyme activity in tumour cells, thereby enhancing their protection against oxidative stress (Figure 5B).

### 2.7. Computational Prediction of Drugability of Cobalt and Vanadium Complexes

Performed drugability assessment shows that all tested complexes met key requirements for a drug candidate. In particular, topological polar surface area (tPSA) values calculated for 1 and 2 cobalt compounds were low (Table 2). All tested complexes had high lipophilicity. The analysis also revealed that complexes 1, 2 and 3 do not express the potency to inhibit the activity of CYP P450 and, overall, the tested substances only slightly violated the Lipinski rule of five [23].

## 3. Discussion

As an extension of our research, here we sought a way to further improve the selectivity of metal scorpionate complexes towards tumour cells. To achieve this goal, we chose complexes containing N,N-bis(3.5-dimethylpyrazol-1-ylmethyl)amine (L^D^), which is an analogue of N,N,N-tris(3.5-dimethylpyrazol-1-ylmethyl)amine (L^S^) that has been investigated previously [6,15,19]. For this study, we prepared three coordination compounds [Co(L^D^)_2_]I_2_∙CH_3_OH (1), [CoL^D^(NCS)_2_] (2) and [VOL^D^(NCS)_2_]∙C_6_H_5_CH_3_ (3) containing dipodal tridentate ligand L^D^ formed in situ from 1-hydroxymethyl-3,5-dimethylpyrazole as a substrate (Scheme 1). In contrast to previous research [6,15], where syntheses of complexes with N,N,N-tris(3.5-dimethylpyrazol-1-ylmethyl)amine were presented, the complexes used in this study contained ligand (L^D^), being a secondary amine, a type of an incomplete scorpion ligand (Scheme 1). This multipodal, tridentate organic ligand formed in situ can be called an “impaired” scorpionate, an analogue of poly(pyrazolyl)borate ligands, containing only two, instead of three, pyrazole groups.

**Figure 6:**
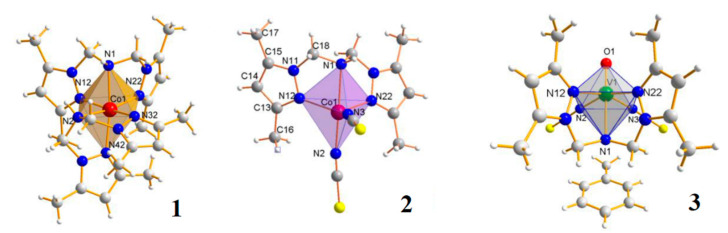
The molecular structures of [Co(L^D^)_2_]I_2_∙CH_3_OH (1), [CoL^D^(NCS)_2_] (2) and [VOL^D^(NCS)_2_]∙C_6_H_5_CH_3_ (3) complexes [18,19,22].

Generally, complexes with an N,N-bis(3,5-dimethylpyrazol-1-ylmethyl)amine (L^D^) ligand are much less numerous than those with tripodal teradentate ligands [16,18,19,22]. Only nine complexes with N,N-bis(3,5-dimethylpyrazol-1-ylmethyl)amine have been reported in CCDC, and therein six complexes were synthesised by us [16,18,19,21,22]. Here, we have tested three selected complexes, containing the same ligand (L^D^), and formed anionic and neutral complexes with different geometries. We particularly attempted to understand how the stoichiometry and structural properties may influence cytotoxic potency and selective action of complexes with multipodal ligands against tumour cells (Figure 6).

All presented complexes (1, 2, 3) contain the organic N,N,N-donor dipodal ligand such as N,N-bis(3,5-dimethylpyrazol-1-ylmethyl)amine (being a secondary amine) and a chelate, dipodal, tetradentate ligand, and form a rigid five-membered ring.

In particular, compound 1 is homoleptic anionic complex with octahedral geometry. A coordination sphere of the cobalt(II) ion in 1 is built by two tridentate organic ligands (L^D^) resulting in *cis-mer*-[Co(L)_2_]^2+^ cation formation (Figure 6). Since an organic ligand possesses two pyrazole N_py_ atoms and one amine N_am_ atom, two isomers are possible for the *mer*-[Co(N_am_)_2_(N_py_)_4_]–type complex. Two amine N_am_-atoms coordinate in *cis* fashion. Such *cis-mer* arrangement of nitrogen atoms leads to a large angular distortion of the cobalt(II) octahedron.

In contrast to 1, complexes 2 and 3 contain one molecule of organic ligand L^D^ and the remaining coordination sites are occupied by monodentate, inorganic ligands (NCS^-^). In 2 and 3, the central ion coordinates five nitrogen atoms (three nitrogen atoms from L^D^ and two from NCS^-^) arranged in a trigonal bipyramidal geometry (2), in contrast to the pseudo-octahedral arrangement of 1 (Figure 6). In all complexes, the order of M-N bond lengths is: M-N_(amine)_ > M-N_(pyrazole)_ > M-N_(NCS)_. The M-N-donor distance and the N-donor–Co–N-donor angles creates the optimal rigid structure, which determines the formation of a stable complex [18,19,22].

It has been reported that cobalt(II) scorpionate complexes may exhibit cytotoxicity in vitro towards HepG2 cells [8]. However, mechanistic studies in terms of on anticancer activity of scorpionate complexes are scarce. In the present study, we found out that similarities and differences in the stoichiometry and the structures of synthesized complexes manifested in diverse interactions with tumour and normal cell membranes. The cobalt complex [Co(L^D^)_2_]I_2_∙CH_3_OH (1) had the highest potency to damage cancer cell membrane (Figure 1B) and expressed the greatest antiproliferative properties towards tumour cells among other complexes (Table 1). The loss of membrane integrity may result in cancer cell death due to necrosis. In line with this, complex 1 was the most effective pronecrotic agent compared to complexes 2 and 3 (Figure 3B) and had the greatest inhibitory effect on cancer cell growth (Figure 2B).

It has been reported that Co(II) octahedral complexes have potent biological activity, including antifungal and antibacterial actions [24]. Harney et al. [25] and Hurtado et al. [26] reported that Co(III) complexes may prevent cancer progression by targeting transcription factors regulating metastatic properties of the cells. Our study showed that Co(II)-containing compounds target vimentin in Hep G2 tumour cells (Figure 4B). In the network of cancer progression stimuli, vimentin is one of the most important proteins; it allows cancer cells to acquire metastatic phenotypes to invade other tissues. A growing body of evidence suggests that vimentin may be a promising molecular target for novel therapies against many types of cancer [27,28].

However, cobalt-containing compounds may act as double-edged weapons in living systems. In particular, the high toxicity of cobalt compounds was also reported toward normal cells [10,11,29]. In the present study, complex 1 had the greatest cytotoxic effect on tumour cells, but, at the same time, it was the most toxic compound toward normal cells. It should be noted that another Co(II)-containing complex [CoL^D^(NCS)_2_] (2) also damaged cancer cell membranes (Figure 1A), exerted antiproliferative effects on tumour cells (Table 1), and decreased growth of cancer cells (Figure 2B). Nevertheless, compound 2 had the weakest cytotoxic effect on normal cells among all tested complexes (Figure 1A) and, unlike complexes 1 and 3, did not disturb growth of normal cells (Figure 2A). We may speculate that the presence of five-coordinated Co(II)s and L^D^ in complex 2 combined with the trigonal bipyramidal geometry (CN = 5) of the molecule may result in improved selectivity toward tumour cells, especially compared to the octahedral geometry of complex 1 (CN = 6). Unlike compound 2, complexes 1 and 3 were characterized by a pseudo-octahedral arrangement (Figure 1), which probably accounted for similar potent cytotoxicity (Figure 1A) and antiproliferative activity (Table 1) of these compounds against normal cells. In line with this, 1 and 3 also inhibited growth on noncancerous cells (Figure 2A). Indeed, we have previously found that vanadium complex 3 may express antiproliferative potency also towards normal cells [22].

It is worth noting that a positive charge of the cationic complex *cis-mer* [Co(L^D^)_2_]^2+^ (1) can favour interaction with cell membrane, and, probably, enhances cytotoxicity of complex 1, which is also in line with the literature [2,30,31]. Both neutral complexes [CoL^D^(NCS)_2_] (2) and [VOL^D^(NCS)_2_]∙C_6_H_5_CH_3_ (3) were less toxic against noncancerous cells than 1. Compound 3, containing VO^2+^ instead Co^2+^ (2), but having the same organic ligand and two monodentate NCS- ions, expressed stoichiometry as 2 and manifested in a toxicity value of less than 1.

**Figure 7:**
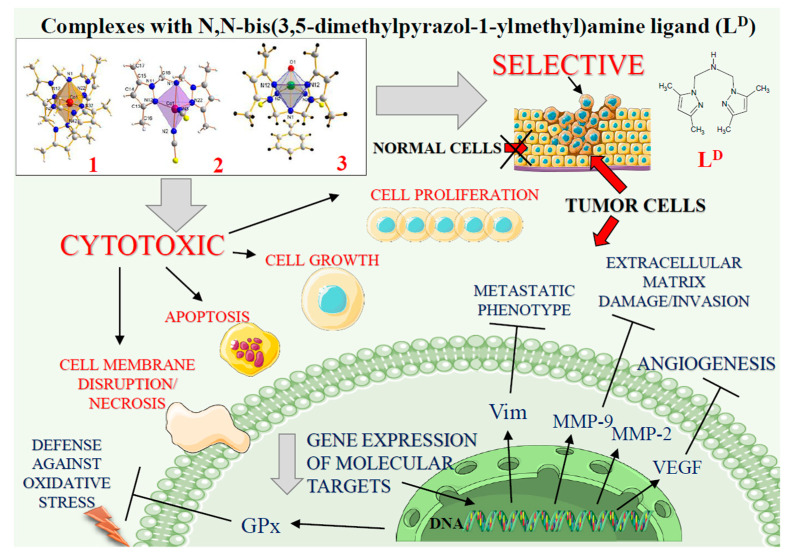
The vanadium and cobalt complexes exert cytotoxicity in tumour cells. Schematic overview of the selective action of complexes 1, 2 and 3 including inhibition of cell viability and disruption of cell membrane. The main actions of compounds on intracellular targets are marked with arrows (Ⱶ inhibition). See text for discussion and abbreviations.

The ability of VO^2+^ complexes to restrain growth of cancer cells has been well-studied using in vitro [8,32] and in vivo [11] models. Anticancer activity of vanadium was also reported in humans [33]. In particular, proapoptotic activity of numerous vanadium compounds has been reported [8,34]. In contrast to necrosis, the initiation of apoptosis is a preferred mechanism of tumour cell death, since in living organisms the elimination of neoplastic cells by such a specific mechanism occurs without inflammation [34,35]. Our data confirm that the presence of VO^2+^ in the structure of complex 3 determined its ability to elicit apoptosis in cancer cells (Figure 3B). Furthermore, the proapoptotic effect of complex 3 was greater when comparing to vanadium salt (VOSO_4_), which underlines the role of complexation vs. the well-established performance of the oxovanadium ion (Figure 3B). What is more, the treatment of tumour Hep G2 cells with complex 3 restrained the expression of specific molecular targets in cancer cells, such as matrix metalloproteinases (MMPs) (Figure 4C,D). MMP-2 and MMP-9 are important therapeutic targets in novel anticancer approaches [36,37]. In particular, the elevated MMP-2 activity during carcinogenesis is associated with angiogenesis and cancer cell migration. Likewise, MMP-9 has been suggested to promote tumour tissue vascularization via vascular endothelial growth factor (VEGF) mobilization [38,39] and our study showed that tested complexes alleviated the expression of VEGF in tumour cells (Figure 7). In particular, the concomitant downregulations of VEGF (Figure 4A) and MMP-2/MMP-9 (Figure 4C,D) by the action of vanadium-containing complex 3 was an exciting finding. At the same time, VOSO_4_ did not exert any effect on MMP-9/MMP-2 expression in tumour cells (Figure 4C,D). Among all the tested complexes, only 3 hampered GPx activity in tumour cells (Figure 5B) and, thus, aggravated their potency to survive. Vanadium compounds administered to animals decreased GPx activity and delayed growth/progression of tumours, as was shown using in vivo models [34]. What is more, complex 3 did not disturb antioxidant enzyme activity in normal cells.

Selective cytotoxicity of complexes 1, 2 and 3, investigated here, were confirmed by computational methods. Drugability assessments of compounds 1, 2 and 3 revealed remarkably low tPSA values for 1 and 2 (Table 2). Thus, both cobalt compounds expressed potency that is easily distributed across membrane barriers in a living organism. In fact, tPSA values lower than 140 Å^2^ are very desirable in new candidates [40]. Additionally, the CYP inhibition potential of new drug candidates is a critical feature at the early stage of drug development process and such activity may be a serious hazard at the stage of preclinical studies [41]. In the current analysis, the potential for all compounds to inhibit CYP was excluded. Taking into consideration several violations from the Lipinski rule of five, all complexes, 1, 2 and 3, met the key requirements for drug candidates (vanadium compound 3 met the criteria to the greatest extent). The lipophilicity of the tested substances was quite high. However, the calculated XLOGP3 values did not exclude them from the group of potential drug candidates [42,43]. Therefore, all compounds should be safe enough to be tested in relevant animal models in the preclinical phase, with introduction of steps appropriate for new chemical entities during drug development process. Summing up, the present study showed that complexes containing N,N-bis(3,5-dimethylpyrazol-1-ylmethyl)amine (L^D^) ligand target cell membranes and regulatory proteins of tumour cells are very promising anticancer compounds worthy of further investigation.

## 4. Materials and Methods

### 4.1. Reagents

All reagents used for the synthesis were purchased from commercial sources and used without further purification. Cobalt powder, NH_4_I/NH_4_SCN, MoO_3_ and 1-hydroxymethyl-3,5-dimethylpyrazole were purchased from Sigma-Aldrich (Seelze, Germany). Elemental analyses were performed with a Perkin-Elmer 2400 CHN elemental analyser and AES-ICP 3410 emission spectrometer (Co) using appropriate Aldrich standards.

### 4.2. Metal Complexes and Their Synthesis

The complexes were prepared according to the general procedure [13,14,20] with some modifications [18,19,22]. Compounds 1 and 2 were isolated from zerovalent cobalt and 1-hydroxymethyl-3,5-dimethylpyrazole (L) as one of the substrates [18,22], whereas 3 was obtained using VOSO_4_. Cobalt powder (325 mesh), 1-hydroxymethyl-3,5-dimethylpyrazole (L), NH_4_I/or NH_4_SCN and MoO_3_ were used in 2:4:6:1 molar ratio for 1 and 2, respectively. Complex 3 was obtained using VOSO_4_·5H_2_O instead of metallic cobalt and the same reagents in the same molar ratio. Isolated compounds are described by the following formulas: [Co(L^D^)_2_]I_2_∙CH_3_OH (1), [CoL^D^(NCS)_2_] (2) and [VOL^D^(NCS)_2_]∙C_6_H_5_CH_3_ (3). The experiments were carried out in an air atmosphere. The products were collected in 42% (1), 65% (2) and 72% (3) yield, respectively. The complexes were air-stable (up to ~100 °C). All complexes were characterized by X-ray diffraction, IR, UV–Vis spectra and thermal investigations [18,19,22]. All isolated complexes contain N,N-bis(3.5-dimethylpyrazol-1-ylmethyl)amine (L^D^) formed in situ from 1-hydroxymethyl-3,5-dimethylpyrazole as a substrate (Scheme 1). The detailed structures of complexes 1–3 are presented in Figure 6.

### 4.3. Cell Culture Experiments

Cell lines were derived from the American Type Cell Culture collection, ATCC (LGC Standards-ATCC, Teddington, Great Britain). ATCC designations were as follows: HEP G2, *hepatocellular carcinoma* cells (HB-8065); CHO-K1, ovary cells (CCL-61). HEP G2 cells were maintained at 37 °C as a monolayer culture in Eagle’s Minimum Essential Medium, EMEM (ATCC). CHO-K1 cells were grown in F-12K medium (ATCC). FBS (10% *v/v*) (Eurx, Gdansk, Poland) was used for media supplementation. In total, 50 µg/mL of gentamicin was added to culture media (Sigma-Aldrich, Seelze, Germany). Trypsin-EDTA solution (Life Technologies, New York, NY, USA) was used for subcultures. Complexes 1, 2 and 3 were dissolved in dimethyl sulfoxide (DMSO, Sigma-Aldrich) prior to cell culture experiments. The cells as well as media were collected after each experiment. The automatic cell counter Countess (Gibco Laboratories, Grand Islands, NY, USA) was used for a precise measurement of cells number. The inverted light microscope Olympus CKX 41SF-5 (Olympus, Germany) was used for the inspection of the morphology of cell culture.

### 4.4. Measurement of Cytotoxic Potency of Complexes with Trypan Blue Exclusion Test

CHO-K1 and HepG2 cells were seeded into 6-well plates (Sarstedt, Numbrecht, Germany) at a density of 2 × 10^4^ cells/well and then incubated for 24 h. The cell monolayers were washed with PBS solution (Sigma-Aldrich) and medium in each well was replaced with a new one, with addition of 50 µM/L of tested compound. Then, the cells were incubated in standard conditions for 24, 48 or 72 h. The cells cultured in the medium with addition of 1% *v/v* DMSO (Sigma-Aldrich) were positive controls (100% of growth). After incubation, the cells were detached using Trypsin-EDTA solution (Life Technologies) and suspended in buffered PBS (Sigma-Aldrich). Trypan Blue Exclusion dye (0.4% *v/v*, Sigma-Aldrich, Germany) was added to each cell culture and samples were kept at room temperature for 5 min. Then, the numbers of live cells (unstained) and dead cells (blue) in each population exposed to tested chemicals/dissolvent were measured with an automatic cell counter.

### 4.5. Cell Growth Assessment

For experiments, the cells were seeded at low density (3 × 10^3^/mL) into the 6-well plates (Sarstedt, Numbrecht, Germany) and kept for 24 h. Then, the medium was replaced for new one, containing 50 µM/L of tested compound or 1% *v/v* of DMSO (control). The time points of incubations were set at 24, 48 or 72 h. At the end of each experiment, the cells were gently detached with Trypsin-EDTA solution (Life Technologies), suspended with appropriate growth medium with addition of 10% *v/v* FBS and centrifuged at 350× *g* for 5 min. Finally, the cells were suspended in buffered PBS (Sigma-Aldrich) and counted [44].

### 4.6. Antiproliferative MTT Assay

MTT proliferation assay (3-[4,5-dimethylthiazol-2yl]-2,5-diphenyl tetrazolium bromide, MTT; Sigma-Aldrich, Seelze, Germany) was performed as described previously [6]. Briefly, cells at density of 1 × 10^5^/mL were seeded on microtiter 96-well plates (BD Biosciences, CA, USA) and incubated overnight. Then, the medium in each well was replaced with a new one, containing the adequate volume of a stock solution of each tested compound (the dilution range was from 10^–3^ to 10^–7^ M/L). The cells cultured in the medium with addition of 1% *v/v* of DMSO were controls (100% of growth). The cells were exposed to the compounds for 72 h. After incubation, the medium was removed and MTT reagent was added to each well. The coloured product of the reaction, MTT formazan, was dissolved in DMSO. The absorbance in each well was recorded at 570 nm (the reference wavelength was 630 nm) using a microplate reader Infinite M200 Pro, Tecan, Austria. IC_50_ values, that indicate antiproliferative potency of tested compounds, were calculated from concentration–response curves (IC_50_ was a concentration of a tested compound (μM/L) required to decrease cell proliferation to 50% of the control) [15]. All data were expressed as arithmetic mean values and standard deviation (M; ±SD).

### 4.7. Apoptosis and Necrosis Detection

For flow cytometry analyses, CHO-K1 and Hep G2 cells were plated in triplicates into 6-well plates (Sarstedt) at a density of 2 × 10^4^ cells per well. The cells were treated for 24 h with tested complexes/comparator salts at concentration of 50 µM/L. Control cells were exposed to DMSO (1% *v/v*)for 24 h. Then, the cells were detached using Trypsin-EDTA solution, washed with buffered PBS (Lonza, Walkersville, MD, USA), centrifuged at 350× *g* for 5 min and suspended in binding buffer. Flow cytometry analysis was performed according to the manufacturer’s instructions (Biotium, Hayward, CA, USA). Fluorescent dyes, 488-AnnexinV (excitation maximum at 490 nm/emission maximum at 515 nm) and/or Ethidium homodimer (EthD-III; excitation maximum at 528 nm/emission maximum at 617 nm) were added to cell suspensions [45]. To correct discrimination between cells and debris, SYTO 41 Blue Fluorescent Nucleic Acid Stain was used (excitation maximum at 483 nm/emission maximum at 503 nm). The cells were incubated in dark for 15 min with appropriate dye/dyes and analysed using flow cytometer FACSCanto10C with BD FACSDiva 8.1 System Software (BD Biosciences Immunocytometry Systems, San Jose, CA, USA). The cells were gated according to forward (FSC), side scatter (SSC) and fluorescence parameters (FITC channel was used for 488-AnnexinV and Texas Red channel was used for EthD-III). The results are given as the percentage of apoptotic or necrotic cells of total counted cells [46].

### 4.8. Measurement of Gene Expression With Real-Time PCR (Quantitative Polymerase Chain Reaction, qPCR)

Hep G2 cells were used to establish cell cultures (2 × 10^4^ cells/well). After 24 h of incubation with chemicals at concentration of 50 µM/L or with 1% *v/v* DMSO (control), the cells were suspended in RL buffer (EURx, Gdansk, Poland) and the total RNA was extracted using Universal RNA purification Kit (EURx, Gdansk, Poland), according to the manufacturer’s protocol. For cDNA synthesis, MMLV reverse transcriptase (Promega, Madison, WI, USA) was used. The reverse transcription reaction was performed with ProFlex PCR System (Applied Biosystems, Foster City, CA, USA). The QuantStudio 7 Flex (Applied Biosystems, Foster City, CA, USA) was employed for the real-time PCR (qPCR). The following Taq-Man human probes (Applied Biosystems) were used for qPCR reaction: VEGFA (Hs00173626_m1), VIM (001855284_m1), MMP-2 (Hs00234422_m1), MMP-9 (Hs00234579_m1) and GAPDH (Hs99999905_m1). Blank qPCR Master Mix (EURx) was used according to the manufacturer’s instructions. Expression data were normalized on the mean housekeeping gene (GAPDH) as a reference. mRNA expressions were obtained with the 2^−ΔΔCt^ method and relative expression values were presented, as previously published [47].

### 4.9. Measurement of Extracellular Glutathione Peroxidase (GPx) Activity

Glutathione peroxidase (GPx) activity was assayed spectrophotometrically in culture medium and enzyme function against reactive oxygen species was investigated according to Adachi et al. [48], Comhair et al. [49] and Howie et al. [50] with modification of Zagrodzki [51], using hydrogen peroxide (H_2_O_2_) as a substrate. For experiment, cell cultures of CHO-K1 and Hep G2 cells were established with initial density of the cells as 2 × 10^4^ cells/well. Then, the cells were exposed to 50 µM/L of each chemical for 24 h (control cells were incubated with 1% *v/v* of DMSO) and cell media were collected. Briefly, the samples were incubated with mixture of sodium azide, glutathione reductase, glutathione, reduced β-nicotinamide adenine dinucleotide phosphate (NADPH) and sodium phosphate (pH 7.6) for 2 min at 25 °C and the reaction was initiated by the addition of H_2_O_2_. NADPH was converted to NADP and the amount of the product was proportional to GPx activity. The decrease in absorbance at 340 nm over 3 min was recorded using a microplate reader Infinite M200 Pro, Tecan, Austria. The mean value was calculated and expressed as U/L of the cell culture medium.

### 4.10. Computational Prediction of Drugability

Drugability assessment was performed with the use of SwissADME software powered by ChemAxon [52,53]. At first, step mol files were translated into Simplified Molecular Input Line Entry Specification by Marvin JS [54]. Calculations were made using build-on equations and algorithms. Key parameters and predictors calculated cover: tPSA—topological polar surface area; XLOGP3—lipophilicity; CYP—cytochrome P450 inhibitor (CYP1A2, CYP2C19, CYP2C9, CYP2D6, CYP3A4) and Lipinski _viol_—number of violations from Lipinski rule of five.

### 4.11. Statistical Analysis

Each experiment was repeated three times. All data are expressed as means ±SD. One-way ANOVA followed by Duncan post-hoc test was used to check for significant differences between groups. Differences were considered significant at *p* values < 0.05. The statistical significance of the obtained data was analysed using the commercially available packages Statistica PL v.10 (StatSoft, Tulsa, OK, USA).

## 5. Conclusions

Herein, selective cytotoxicity of complexes [Co(L^D^)_2_]I_2_∙CH_3_OH (1), [CoL^D^(NCS)_2_] (2) and [VOL^D^(NCS)_2_]∙C_6_H_5_CH_3_ (3) were studied in tumour cells. The complexes (1), (2) and (3) containing N,N,N-donor dipodal ligand such as (N,N-bis(3,5-dimethylpyrazol-1-ylmethyl)amine were more active and more selective than their salts (CoCl_2_, VOSO_4_). Thus, the complexation process was the favourable factor that enhanced anticancer properties of the compounds. Additionally, coordination numbers (CNs) of metal ions, imposing specific shapes to molecules, contributed to biological properties of investigated complexes. All tested complexes containing dipodal tridentate N,N-bis(3,5-dimethylpyrazol-1-ylmethyl)amine (L^D^) ligand expressed greater anticancer activity and were less toxic toward noncancerous cells than cisplatin, which is widely used in clinical practice. Calculated AI index showed that metal complexes with N,N-bis(3,5-dimethylpyrazol-1-ylmethyl)amine (L^D^) expressed great selectivity towards tumour cells. Additionally, computational prediction of drugability of compounds 1, 2 and 3 met the key requirements for a drug candidate. This is a promising result regarding further modifications and optimisations of these compounds.

## Figures and Tables

**Table 1:** IC_50_ values (μM) of tested compounds when inhibiting the metabolic activities and proliferation of noncancerous cells (CHO-K1) and tumour cells (Hep G2), as determined by the MTT assay. Results are arithmetic mean values and standard deviation (M; ±SD), *n* = 3. Data adapted from previous study [15,22] are denoted in the table.

Compound	IC_50_ [μM] for Noncancerous CHO-K1 Cells	IC_50_ [μM] for Cancer HEP G2 Cells	Antiproliferative Index IC_50_ CHO-K1/IC_50_ HEP G2
1	121.8 ± 10	22.0 ± 4	5.5
2	268.2 ± 15	38.2 ± 5	7.0
3	123.1 ± 3	45.6 ± 5	2.7
VOSO_4_	98.3 ± 9 [22]	75.6 ± 8	1.3
CoCl_2_	175.6 ± 28 [15]	160.2 ± 18 [15]	1.1
Cisplatin	19.1 ± 2	21.3 ± 2 [15]	0.9

**Table 2:** Drugability assessment of complexes 1, 2 and 3 based on structure–activity relationship analysis (tPSA–topological polar surface area; XLOGP3—lipophilicity; BBBperm—blood–brain barrier permeability; Pgpsub—p glycoprotein substrate; CYP—cytochrome P450 inhibitor (CYP1A2, CYP2C19, CYP2C9, CYP2D6, CYP3A4); Lipinski_viol_—number of violations from Lipinski rule of five).

Compound	tPSA [Å^2^]	XLOGP3	CYP_inh_	Lipinski_viol_
1	45.36	14.43	No	3
2	37.95	9.03	No	2
3	146.9	6.3	No	1

## Data Availability

The data presented in this study are available on request from the corresponding author.
